# Targeting of *PHOX2B* expression allows the identification of drugs effective in counteracting neuroblastoma cell growth

**DOI:** 10.18632/oncotarget.19922

**Published:** 2017-08-04

**Authors:** Eleonora Di Zanni, Giovanna Bianchi, Roberto Ravazzolo, Lizzia Raffaghello, Isabella Ceccherini, Tiziana Bachetti

**Affiliations:** ^1^ U.O.C. Genetica Medica, Istituto Giannina Gaslini, Genova, Italy; ^2^ Laboratorio di Oncologia, IRCCS G. Gaslini, Genova, Italy; ^3^ Department of Neurosciences, Rehabilitation, Ophthalmology, Genetics, Maternal and Child Health and CEBR, Università degli Studi di Genova, Genova, Italy; ^4^ Present Address: Istituto di Biofisica, CNR, Genova, Italy

**Keywords:** PHOX2B, ALK, neuroblastoma, gene expression regulation, drug screening

## Abstract

The pathogenic role of the *PHOX2B* gene in neuroblastoma is indicated by heterozygous mutations in neuroblastoma patients and by gene overexpression in both neuroblastoma cell lines and tumor samples.

*PHOX2B* encodes a transcription factor which is crucial for the correct development and differentiation of sympathetic neurons.

*PHOX2B* overexpression is considered a prognostic marker for neuroblastoma and it is also used by clinicians to monitor minimal residual disease. Furthermore, it has been observed that neuronal differentiation in neuroblastoma is dependent on down-regulation of *PHOX2B* expression, which confirms that PHOX2B expression may be considered a target in neuroblastoma.

Here, *PHOX2B* promoter or 3′ untranslated region were used as molecular targets in an *in vitro* high-throughput approach that led to the identification of molecules able to decrease *PHOX2B* expression at transcriptional and likely even at post-transcriptional levels. Further functional investigations carried out on *PHOX2B* mRNA levels and biological consequences, such as neuroblastoma cell apoptosis and growth, showed that chloroquine and mycophenolate mofetil are most promising agents for neuroblastoma therapy based on down-regulation of *PHOX2B* expression.

Finally, a strong correlation between the effect of drugs in terms of down-regulation of *PHOX2B* expression and of biological consequences in neuroblastoma cells confirms the role of *PHOX2B* as a potential molecular target in neuroblastoma.

## INTRODUCTION

Neuroblastoma is the most frequent pediatric extracranial solid tumor accounting for 15% of all child deaths from cancer. It's caused by aberrant proliferation of undifferentiated neural crest cell progenitors in the developing sympathoadrenal lineage of the nervous system. In the last decade, several studies showed a role of genetic factors in neuroblastoma through the identification of several susceptibility loci by genome-wide association studies [1–2] and the detection of somatic mutations by next generation sequencing [2] and of chromothripsis in up to 18% of high-risk neuroblastomas [3].

Since 2004, the paired-like homeobox 2B (*PHOX2B*) gene, encoding a transcription factor crucial for the early steps of autonomous nervous system development, proved to take part in the complexity of the genetic landscape of neuroblastoma. Indeed, heterozygous mutations of the *PHOX2B* coding region were identified in sporadic and familial cases of isolated or syndromic neuroblastoma, a tumor associated with other neurocristopathies such as congenital central hypoventilation syndrome and Hirschsprung disease [4–6].

In addition to rare heterozygous *PHOX2B* mutations detected in neuroblastoma, the pathogenetic role of this gene in neuroblastoma is also indicated by its anomalous overexpression in tumor samples and cell lines, which correlates with the excessive expression of its transcriptional target *ALK* [7]. High *PHOX2B* mRNA levels are therefore considered a sensitive prognostic marker for neuroblastoma since, at diagnosis, they identify children with ultrahigh-risk disease [8]. Furthermore, the evaluation of *PHOX2B* gene expression is currently used to monitor minimal residual disease in neuroblastoma patients [9].

*In vivo* studies showed that *Phox2B* is down-regulated during the neuronal differentiation process and that *Phox2B* overexpression plays a crucial role in the arrest of neuronal differentiation in TH-MYCN mice [10]. Accordingly, high expression levels of *Phox2B* promote neuroblastoma cell proliferation and xenograft tumor growth, while proliferation of undifferentiated *Phox2B* expressing neuronal progenitors is suggested as a mechanism inducing neuroblastoma development [11]. Consistently, retinoic acid-induced neuronal differentiation is dependent on down-regulation of *PHOX2B* expression, which confirms the pathogenic role of *PHOX2B* excessive levels [12]. The predominant cells in both hyperplastic lesions and in neuroblastoma samples are largely represented by Phox2B+ progenitors, whose number correlates with tumor growth [10], which suggests a PHOX2B role also in tumor progression. However, very recent data suggest that *PHOX2B* overexpression is likely pathogenic in the earliest steps of neuroblastoma growth, associated with poor neuroblast differentiation and expansion, while in the final metastatic phase it seems to protect against aggressive migration capability [13].

Therefore, as correct levels of *PHOX2B* are crucial for a proper neural differentiation, *PHOX2B* gene expression can be considered a druggable target against neuroblastoma development. Indeed, the beneficial effect of *PHOX2B* transcriptional down-regulation by curcumin, SAHA and trichostatin A, alone or in combination, has been recently reported by us in terms of mRNA decrease in both *PHOX2B* and its transcriptional target *ALK* [14]. This data was further confirmed by the observation that the differentiating effect of all-transretinoic acid (ATRA) is mediated by down-regulation of *PHOX2B* transcription in neuroblastoma cells [15].

Functional and genetic studies demonstrated that *PHOX2B* expression is regulated also at post-transcriptional levels. In particular, we showed that miR-204 down-regulates *PHOX2B* mRNA by acting on a specific 3′UTR element [16]. Such observation indicates that the complete absence or the presence of very low miR-204 levels in neuroblastoma samples [17] is responsible to some extent for excessive *PHOX2B* expression in this tumor.

Here, by using an already described experimental approach [14], we report the screening of a library of 640 Food and Drug Administration (FDA) approved molecules, aimed at searching for compounds able to decrease *PHOX2B* expression in neuroblastoma cells through regulation of gene transcription and/or mRNA stability.

## RESULTS

### High throughput drug screenings

**Figure 1:**
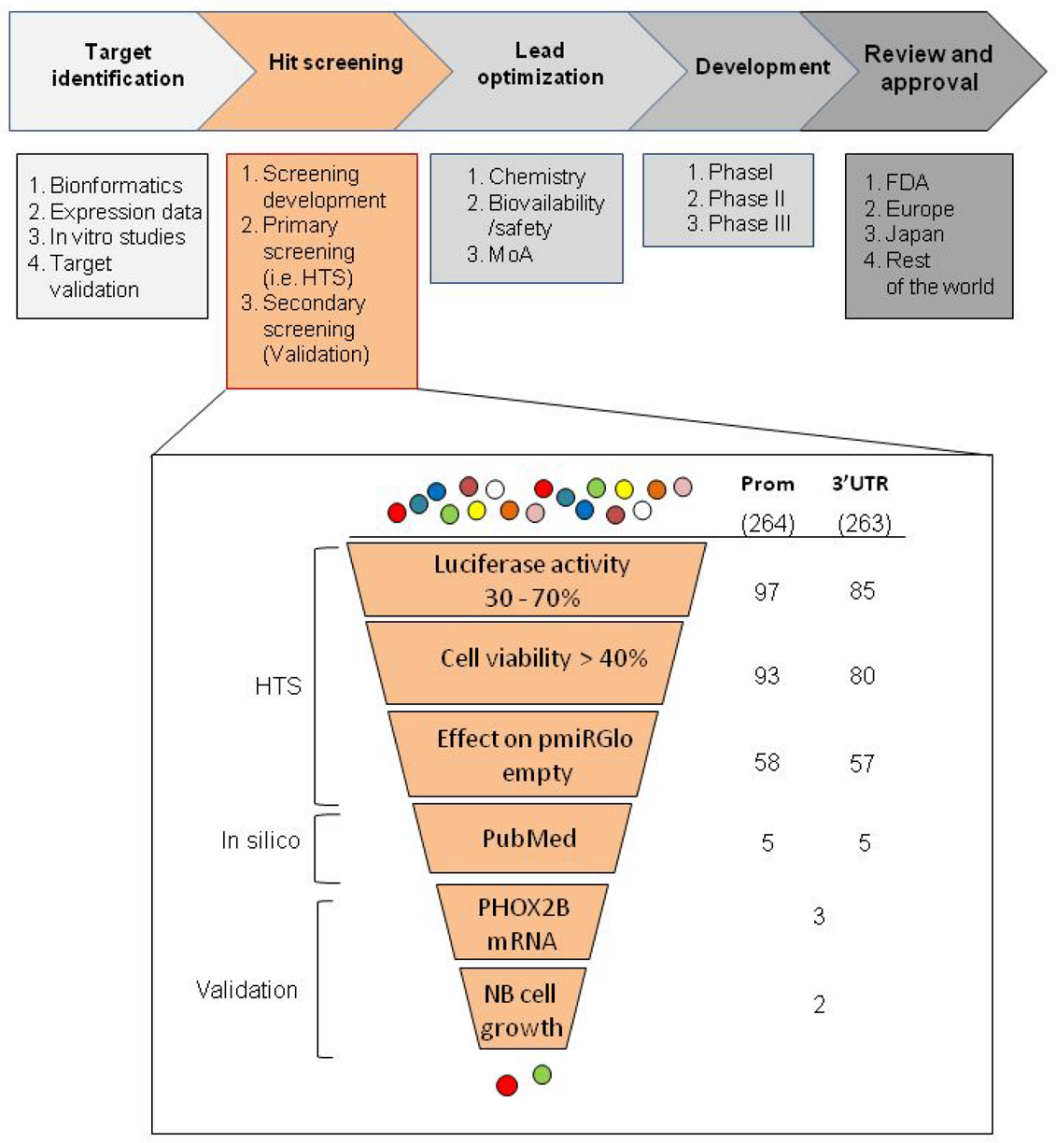
Workflow of the selection criteria applied in the high throughput drug screenings In the upper part of the figure, the schematic representation of the phases in which a drug discovery process can be subdivided is reported, each of them containing several steps. In the lower part of the figure, a zoom in the “hit screening” phase is shown to list the selection criteria used in this work to obtain a discrete number of drugs for validation. In particular, we have listed the selection criteria used to obtain an appropriate number of drugs to be validated. In the zoomed image, on the left side criteria applied for the automated screening of the molecules are collectively defined “HTS”, criteria using information already reported in the literature are defined “Pubmed”, and criteria based on biological assays are defined “validation”. Each method applied is indicated in the near boxes, whose progressively smaller dimensions represent the amount of drugs selected in each step. On the right, the initial number of molecules effective following normalization on viability is indicated for both promoter and 3′UTR HTS assays (264 and 263, respectively). Below, in the two columns, the residual molecules filtered after each selection are indicated.

Based on previous data suggesting *PHOX2B* overexpression as a possible molecular target for neuroblastoma therapy, we performed two high throughput screenings (HTS) of drugs following a standard drug discovery process, which can be classified into five steps, namely: i. target identification, ii. hit screening, iii. lead optimization, iv. development, v. review and approval (Figure 1, upper image). While we focused on “target identification” in previous studies [7, 14], we have recently approached the second step of the process, namely “hit screening”.

Within this step, the experimental plan of our work has consisted in the following three phases: 1. a pilot study to set up the experimental conditions (screening development); 2. HTS (primary screening); 3. *in vitro* validation (secondary screening).

First, DMSO tolerance and best performing combination of DMSO (as drug solvent) and drug concentrations were assessed to avoid negative effects on cell viability and Luciferase activity. In particular, a IMR32 clone stably transfected with the *PHOX2B* promoter driving the *luciferase* gene expression was added with increasing doses of trichostatin A (TSA) and triacetyl-resveratrol from 100nM to 20μM, previously selected as positive and negative controls of the assay, respectively [14]. By merging results of DMSO tolerance (Supplementary Figure 1A) with effects of control drugs (Supplementary Figure 1B), we decided to perform HTS at 5 μM drug concentration in 0.5% of DMSO.

**Figure 2:**
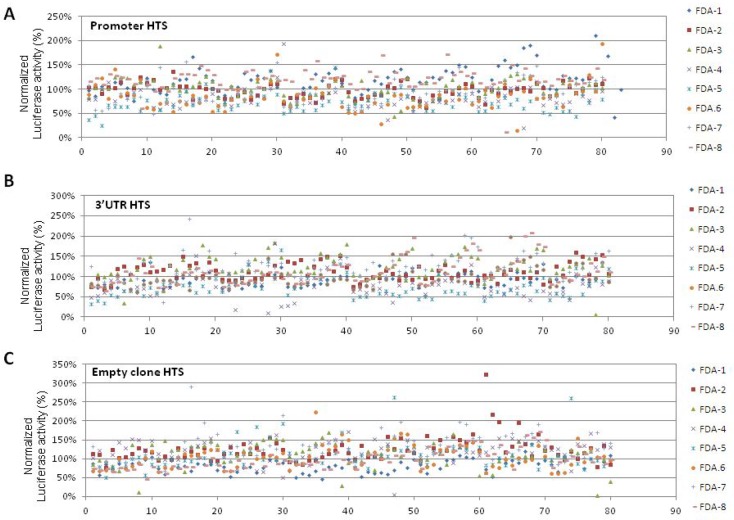
Schematic representation of drug effects in “Promoter HTS”, “3′UTR HTS”, and “Empty HTS” The effects of the drugs in the three cellular models is represented in terms of percentage of Luciferase activity normalized on cell viability (Y axis), normalized on the 100% threshold defined as the value obtained in only DMSO treated cells. Drugs are numbered from 0 to 80 (X axis) and indicated with 8 different colored symbols, one for each multiwell plate numbered FDA 1-8 on the right side (see Legend). Diagrams represent results obtained in the three HTS performed on the PHOX2B promoter “Promoter HTS” (**A**), on the PHOX2B 3′UTR “3′UTR HTS” (**B**), and on the empty clone “empty HTS” (**C**).

In order to perform drug repositioning, two HTS of 640 FDA-approved compounds, i.e. a collection of molecules with known and well-characterized bioactivity, safety, and bioavailability, were performed to investigate the effect of molecules on the *PHOX2B* promoter and the 3′untraslated region, hereon defined “promoter HTS” and “3′UTR HTS”. In particular, IMR32 cells were treated with 5 μM compound concentration and were tested for Luciferase activity and cell viability after 24 h of drug exposure. Moreover, in each plate, in addition to empty and DMSO only wells, a total of eight samples were repeatedly treated with trichostatin A (4) and triacyl-resveratrol (4) [14]. The activity of the *PHOX2B* promoter/3′UTR was calculated on the basis of Luciferase values in drug-treated samples, expressed as percentage of the vehicle only treated cells (DMSO) and then normalized on cell viability. Such normalization of results allowed us to abandon drugs whose effects could be only ascribed to a variation of the cell number (Figure 2A–2B).

In “3′UTR HTS”, to minimize false positive drugs acting on the backbone of the integrated vector or on the promoter driving the *luciferase* gene expression, independently of the *PHOX2B* 3′UTR, we tested the library in duplicate also on an IMR32 clone stably transfected with the empty pmiRGlo vector, expressing the *luciferase* reporter under the control of the PGK promoter and controlled downstream by its polyA sequence (Figure 2C). Moreover, to select drugs specifically acting on the *PHOX2B* promoter in the “promoter HTS”, we used the above information also to exclude drugs likely acting on the PGK promoter. Overall, 264 and 263 molecules resulted able to reduce Luciferase activity as a consequence of down regulation of the *PHOX2B* promoter and of the 3′UTR region, respectively, some of them acting on both regions. To select a discrete number of molecules to validate, we applied filters based on the following criteria (Figure 1, lower image):

1. To exclude too low or too high Luciferase activities, likely representing potential biases in drug assessment, in both promoter and 3′UTR HTS molecules were considered only with Luciferase values between 30–70% normalized on untreated (DMSO only added) cells; 2. to avoid excessive toxic effects that may have impaired Luciferase activity, only drugs inducing cell viability (considered as the percentage of living cells, not killed by drug treatment, with respect to DMSO added cells) higher than 40% were considered for further investigation.

3. to increase stringency and avoid “ubiquitous” drugs regulating the transcription of the PGK promoter (in the promoter HTS) or *luciferase* gene stability by acting on the SV40 polyA signal (in the 3′UTR HTS), we excluded molecules affecting Luciferase activity of the clone carrying the pmiRGlo empty vector.

We thus obtained 58 molecules from the promoter and 57 molecules from the 3′UTR screenings. Among these drugs, we focused on molecules already reported to have an effect on neuroblastoma or other tumors or to have a role in cellular differentiation (5 selected from promoter and 5 from 3′UTR screenings). As some of these molecules were selected in both HTS, in the end our screening detected a total amount of six different promising compounds (Table 1 [18–27]), namely: lovastatin, acting only on the *PHOX2B* promoter, tranylcypromine, acting only on the 3′UTR, sulindac, acetylsalicylic acid, chloroquine and mycophenolate motefil, acting on both regions. In particular, in terms of activity reduction in one or both *PHOX2B* regulatory regions, we observed that acetylsalicylic acid, chloroquine and mycophenolate showed progressively higher efficacy.

**Table 1:** Drugs selected to undergo validation

Drugs selected	Acronym	Promoter	3′UTR	PubMed NB	PubMed other cancers
LOVASTATIN	LOV	69 ± 12	no	Girgert et al, 1999	Dimitroulakos, 2001
TRANYLCYPROMINE	TRAN	no	49 ± 1		Zheng, 2016
SULINDAC	SUL	56 ± 11	63 ± 0,1		Chan, 2013
ACETYLSALICILIC ACID	AA	51 ± 9	54 ± 6	Carlson, 2013	Chan, 2013
CLOROQUINE PH	CQ	52 ± 8	43 ± 4	Aveic, 2016	Zhang, 2015
MYCOPHENOLATE MOFETIL	MMF	38 ± 3	33 ± 3	Messina 2004 (a,b)	

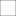
 Activity > 60% or effect only on one regulatory region

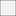
 Both regions with activity [50%–60%]

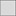
 One of the two regions with activity < 50%

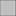
 Both regions with activity < 50%

From the top to the bottom, drugs have been ordered following their ability to reduce Luciferase activity on one or both regions, with higher extent of efficacy shown by increasing grey intensity. From left to right, the acronymous for each drug, the Luciferase activity on promoter and 3′UTR and references reporting their effects on NB or other cancers, are shown.

### Effects of drugs on *PHOX2B* gene expression

In order to confirm that modulation of *PHOX2B* promoter and/or 3′UTR regulatory regions truly results in *PHOX2B* gene expression regulation, selected drugs were tested for their effects on *PHOX2B* mRNA.

**Figure 3:**
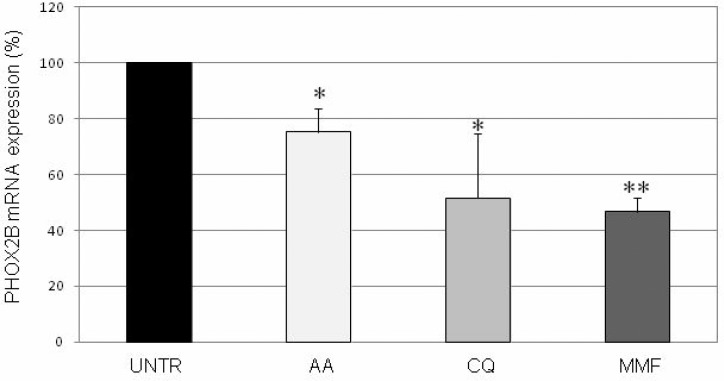
Evaluation of drug effects on PHOX2B mRNA expression Levels of mRNA of endogenous *PHOX2B* in IMR32 following treatments for 24 h with drugs, represented as mean values normalized on both G3PDH and b2m housekeeping genes Grey intensity scale of the bars represents increasing levels of drug effect, in agreement with the grey scale used for Luciferase activity, thus reflecting data reported in Table 1. Values are the mean of three independent experiments ± SD performed in triplicate. Asterisks (*) indicate significant differences compared to DMSO treated cells (Student's *t* test, *p* < 0.05).

To this end, we treated IMR32 native cells with each drug for 24 hours. While tranylcypromine, lovastatin, and sulindac did not show any effect on *PHOX2B* mRNA (not shown), acetylsalicylic acid, chloroquine, and mycophenolate mofetil induced a significant decrease in the *PHOX2B* mRNA expression compared to cells treated with vehicle only (H_2_O or DMSO, depending on the drugs) defined “UNTR” (Figure 3), thus confirming the consistency of the effect produced on the *PHOX2B* regulatory regions. Interestingly, the effect of the three compounds, represented by a progressively stronger effect on *PHOX2B* mRNA starting from AA to MMF, is consistent with their position in the ranking list based on the percentage of Luciferase decrease in one or both regions (see Table 1). On the contrary, the other three drugs reported in Table 1 did not display any effect on *PHOX2B* mRNA, thus suggesting they were false positives or, alternatively, their effect on the promoter and 3′UTR fragments used as molecular targets in HTS cellular models may have been compensated by other flanking regions of the endogenous *PHOX2B* gene displaying opposite effects.

### Evaluation of drug effects on neuroblastoma cell growth

**Figure 4:**
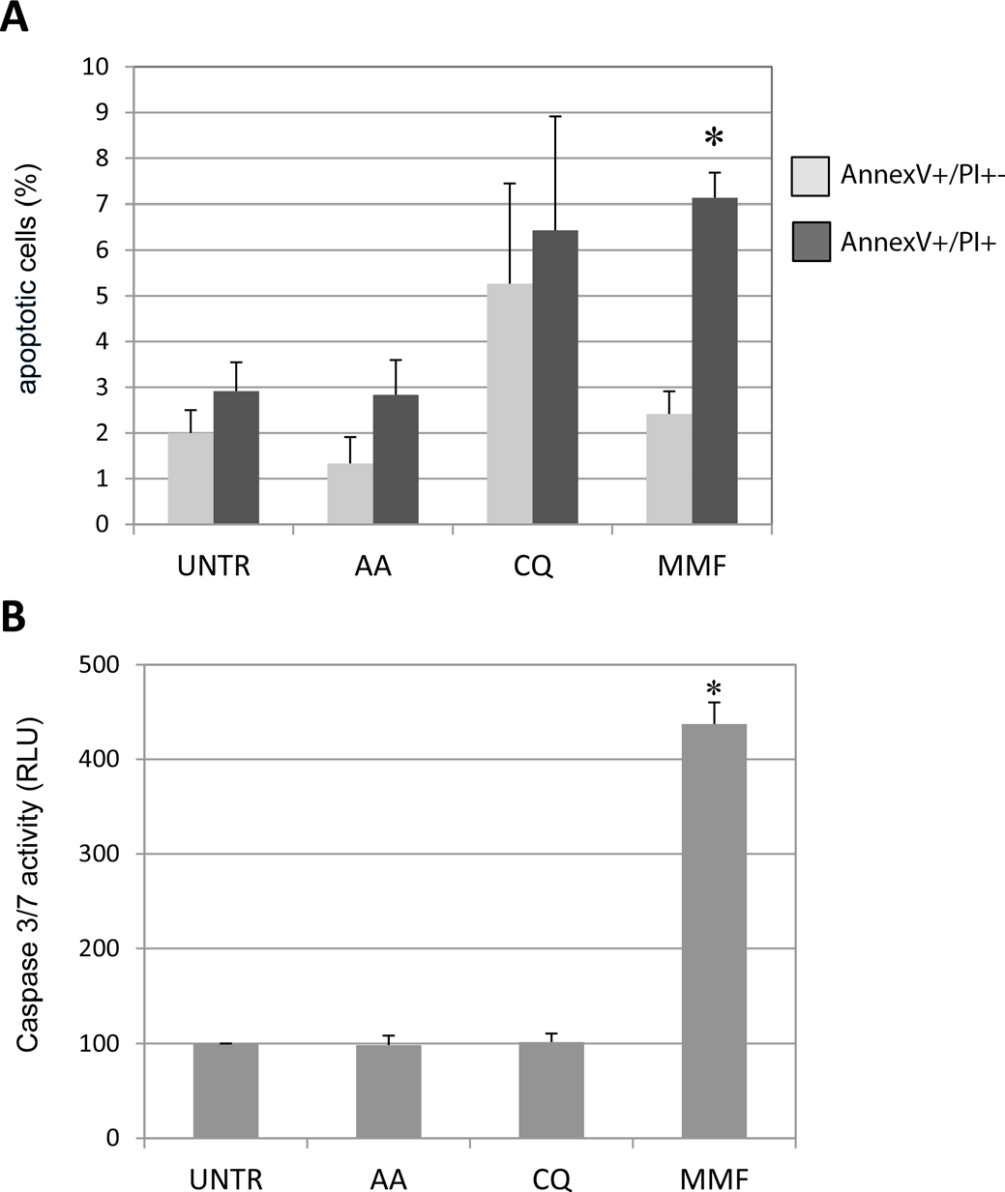
Effect of drugs on apoptosis in neuroblastoma IMR32 cells Staining of IMR32 cells by flow citometry with Annexin V/PI to evaluate apoptosis following 48 h drugs treatments. (**A**) Early (Annexin V+/PI−) and late (Annexin V+/PI+) apoptotic cells, expressed as percentage out of the total number of acquired cells. (**B**) Evaluation of caspase 3/7 activity, expressed as percentage compared to untreated - UNTR cells (DMSO or H2O, depending on the drug), arbitrarily shown as 100%. Values are the mean of three independent samples ± SD; asterisks (*) indicate significant differences compared to untreated cells (Student's *t* test and ANOVA test, *p* < 0.05).

As *PHOX2B* overexpression was suggested to have a role not only in impairing neuronal differentiation but also in neuroblastoma progression [11], we evaluated whether the effects of the selected molecules could interfere with neuroblastoma cell survival, which is the result of the balance between proliferation, cell cycle, and apoptosis. First, in IMR32 cells we evaluated the morphology of nuclei in the presence of each treatment to search for elements suggesting cell death. After 48 hours of treatment, while AA did not produce any effect in terms of nuclear morphology compared to untreated (H_2_O added) cells, MMF treated cells, and to a less extent, also CQ treated cells were characterized by a fraction of aberrant nuclei with fragmented or irregular borders, that are morphological features suggesting apoptosis. (Supplementary Figure 2A–2B). To confirm that aberrant nuclear morphology was associated with apoptosis, we investigated the presence of annexin V on treated cell surface. As shown in Figure 4A and Supplementary Figure 3, 48 hours of MMF treatment was able to induce late apoptosis, as confirmed by the double staining for annexin V and propidium iodide, and by the evaluation of the activity of pro-apoptotic caspases 3 and 7, the effector members of the caspase family responsible for the cleavage of target proteins down-stream the activation of the apoptotic signaling, and thus considered as markers of late apoptosis. In particular, by an approach based on caspase-3/7 cleavage of a luminogenic substrate, we observed that MMF was able to increase the activity of the two apoptotic enzymes, while AA and CQ did not show any effect (Figure 4B). Due to the high variability among neuroblastoma cells, we investigated apoptosis also in HTLA-230; this human neuroblastoma cell line was selected as it already proved to mimic both tumorigenesis and progression in mouse models of neuroblastoma [28], and observations in HTLA-230 could be useful for a potential *in vivo* study. In this cell line, MMF showed a pro-apoptotic effect after 48 h, though not statistically significant. Moreover, in HTLA-230, CQ did not induce apoptosis at all, showing a poor “protective” effect represented by a lower percentage of early apoptotic cells with respect to untreated sample, while cells treated with AA showed an increase in both early and late apoptosis, though not statistically significant. (Supplementary Figure 4A and Supplementary Figure 3).

**Figure 5:**
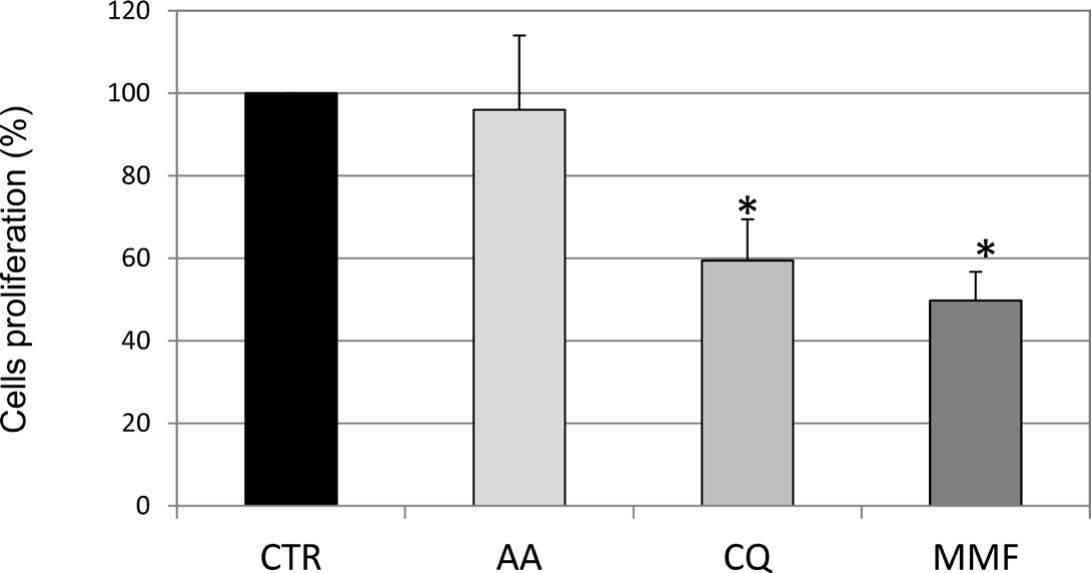
Evaluation of cell proliferation rate following drug treatments Cell proliferation rate after drug addition for 48 h is shown as proliferation rate calculated as 100- inhibition rate (obtained as mean fluorescent intensity-MFI of CFSE), based on the observation that an increase in MFI indicates inhibition of cell proliferation, as dividing cells contain half fluorescent dye. Values are the mean of three independent samples ± SD; asterisks indicate significant differences compared to untreated cells (Student's *t* test, *p* < 0.05). (Grey intensity scale represents increasing levels of drug effect in terms of Luciferase activity, as reported in Table 1).

We then investigated whether the three analyzed drugs were able to interfere with cell growth. In particular, to assess the proliferation rate, IMR32 cells were incubated with CFSE, that labels intracellular molecules. Since when a CFSE-labeled cell divides, its progeny contains half the amount of fluorescence, cell proliferation rate is inversely proportional to cell fluorescence. Therefore, an increase in fluorescence in treated cells compared to untreated cells is suggestive of inhibition of proliferation. Evaluation of IMR32 cell proliferation suggested that MMF and CQ induced cell cycle arrest, while AA was quite ineffective in decreasing neuroblastoma cell proliferation (Figure 5). In HTLA-230 drugs showed a similar effect, less variable for AA although not significant, and less marked for CQ. MMF confirmed a marked effect also in HTLA-230 cells (Supplementary Figure 4B). Overall, while AA and CQ showed different effects on apoptosis and cell proliferation, depending on the neuroblastoma cell line used, MMF showed a similar effect in the two cell lines. Overall, a part from a pro-apoptotic effect shown by AA only in HTLA-230, this drug did not seem effective in counteracting *in vitro* neuroblastoma cell growth. On the other hand, CQ was able to decrease significantly only cell proliferation in both cell lines, while MMF proved effective in both processes in both cell lines. These results allowed us to exclude AA from further investigation and to consider MMF as the most effective molecules among those analyzed.

### Analysis of drug effects on PHOX2B protein levels

**Figure 6:**
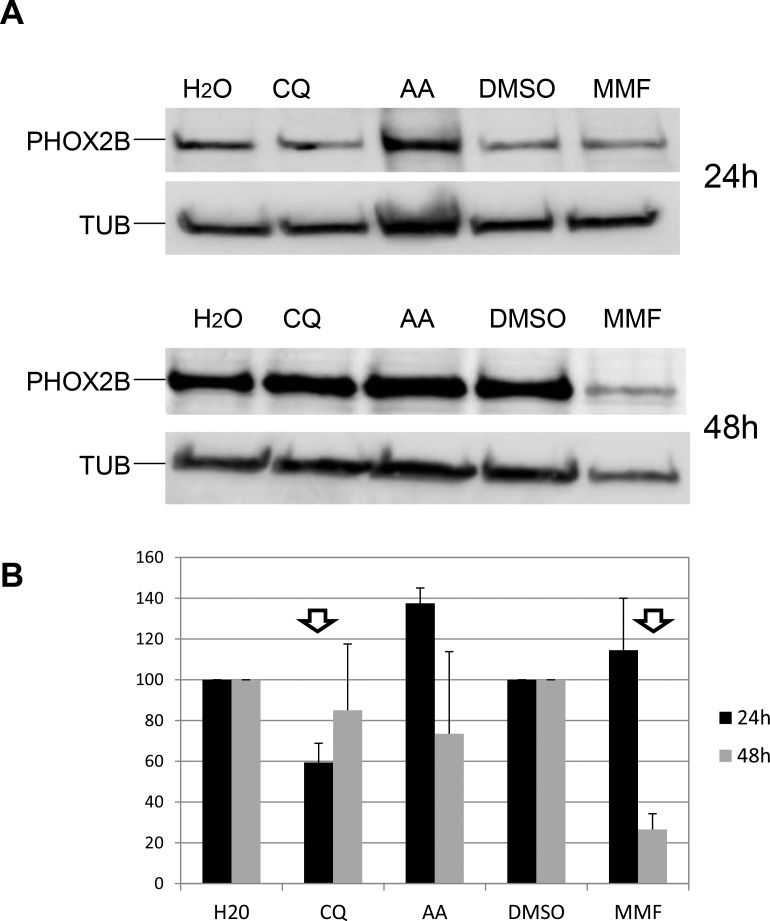
Effect of drugs on PHOX2B protein levels (**A**) Representative image of Western Blot experiments. Lysates from IMR32 cells treated for 24 h and 48 h with AA, CQ and MMF were run on a 10% acrylamide gel electrophoresis. Overnight incubation with antibodies specific for PHOX2B and housekeeping tubulin (TUB) allowed to detect 38 kDa and 50 kDa bands, respectively. H_2_O represents the untreated sample for AA and CQ, DMSO is the untreated sample for MMF. (**B**) The bar diagram shows mean values of PHOX2B levels (%) after drug treatments, expressed as percentage compared to the corresponding untreated sample, defined as 100% (*N* = 2). Arrows indicate effective drugs.

To assess whether differences in drug efficacy could be ascribed to different PHOX2B protein levels, we investigated whether the decrease in PHOX2B mRNA following drug treatments resulted in a reduced PHOX2B protein amount (Figure 6A). In particular, western blot assays performed on IMR32 lysates followed by quantification of signals showed that CQ treatment for 24 h was able to decrease PHOX2B protein while CQ 48 h treatment brought protein amount to normal level. MMF proved effective after 48 h treatment while AA induced an unexpected increase in PHOX2B protein in the first 24 hours of treatments, that disappeared after 48 h prolonged treatment (Figure 6B). Overall, these results show the efficacy of the three drugs on PHOX2B protein level, thus suggesting a correlation between mRNA decrease and downstream effect and confirming MMF as a most promising compound.

### Investigation of drugs effect on *ALK* gene expression

**Figure 7:**
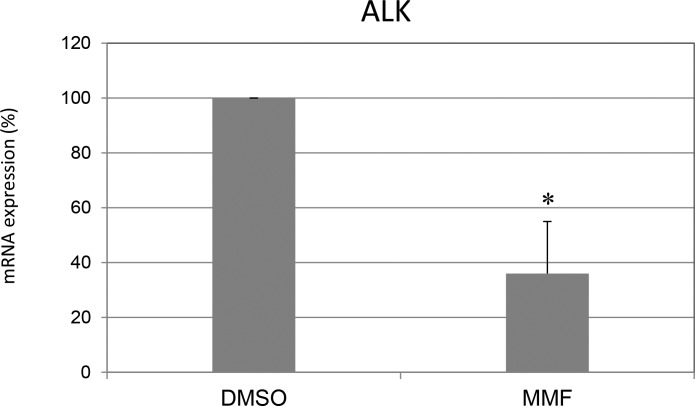
Mycophenolate mofetil effect on ALK mRNA expression Levels of mRNA of endogenous *ALK* in IMR32 following treatments for 24 h with MMF. Values are the mean of three independent experiments ± SD and represent the mean values normalized on both G3PDH and b2m housekeeping genes and compared to untreated (DMSO) cells, defined as 100%. Asterisks indicate significant differences compared to DMSO treated cells (Student's *t* test, *p* < 0.05).

Finally, we wondered whether PHOX2B protein decrease consequent to CQ and MMF treatment could result in down-regulation of Anaplastic Lymphoma Kinase (*ALK*) gene expression, whose transcription is PHOX2B-dependent and overexpression is considered pathogenic in neuroblastoma [7]. As shown in Figure 7, gene expression analysis in IMR32 cells treated for 24 hours showed that MMF was able to decrease *ALK* mRNA levels, thus strengthening the beneficial effect of this molecule, which approved to have a beneficial widespread action. On the contrary, CQ did not show any effect (not shown).

### Quantification of the biological effects of drugs

**Figure 8:**
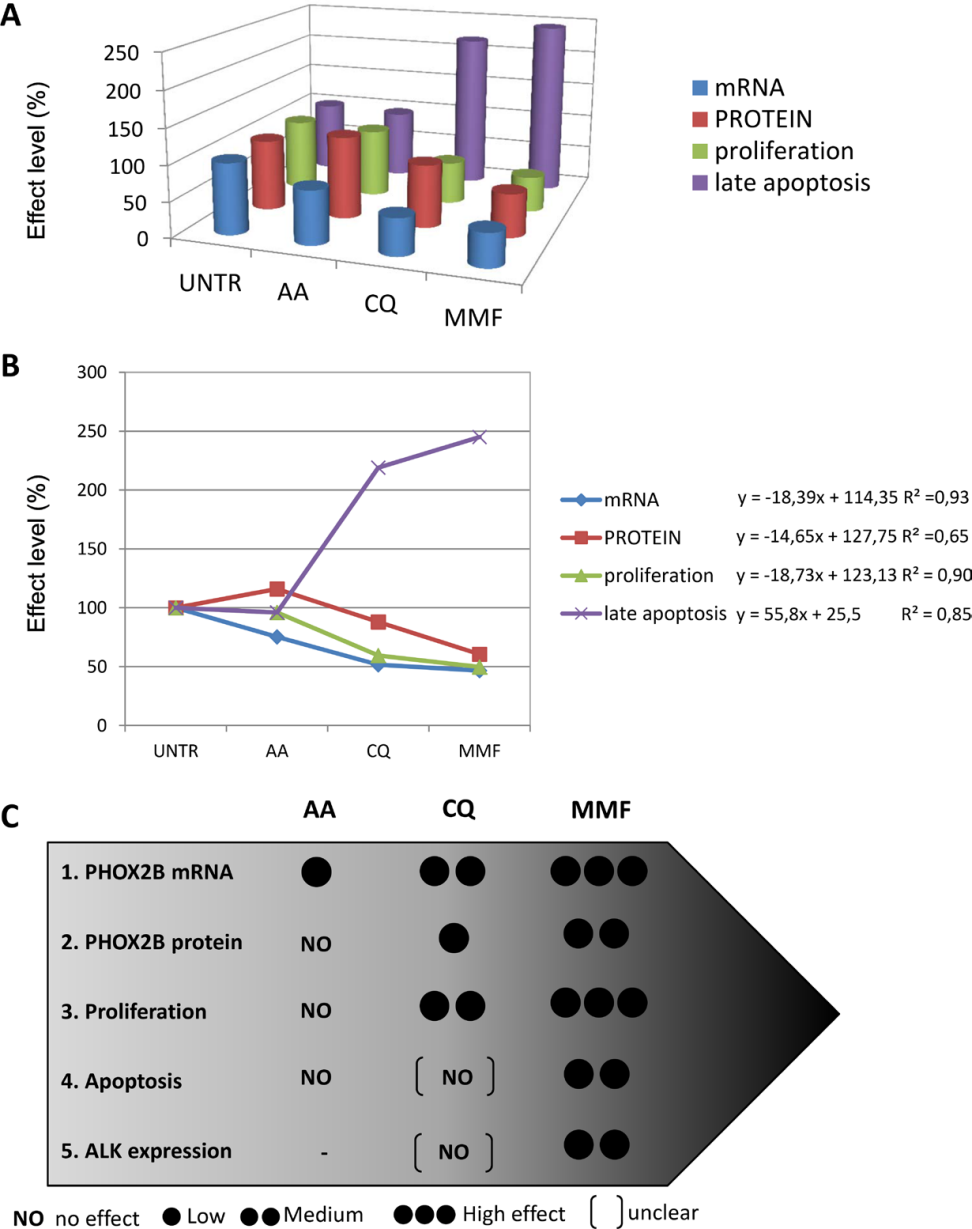
Relation among the effects produced by the three drugs (**A**) The 3D diagram shows all together the mean values of the effects produced by each drug, calculated as percentage of untreated cells (defined as 100%), for each variable: mRNA (blue), protein (red), proliferation (green), apoptosis (violet). Visible although not quantified correlation among the variables is shown. UNTR: DMSO or H2O; AA: acetylsalicilic acid, CQ: chloroquine phosphate, MMF: mycophenolate mofetil. (**B**) The diagram shows the distribution of the values (y-axis), expressed as % compared to UNTR (= 100), of the three treated conditions AA-CQ-MMF (x-axis) for each variable: mRNA (blue), protein (red), proliferation (green), apoptosis (violet); UNTR: DMSO or H2O, depending on the drug; AA: acetylsalicylic acid, CQ: chloroquine phosphate, MMF: mycophenolate mofetil. For each variable, the linear function and the coefficient of regression are also shown on the right side of the graph. (**C**) Graphical representation of the efficacy of the three drugs. The five biological effects (1. down-regulation of *PHOX2B* mRNA, 2. down-regulation of PHOX2B protein, 3. inhibition of proliferation, 4. induction of apoptosis, 5. decrease in *ALK* expression) were considered for each of the three drugs (AA, CQ and MMF). The number of black circles is proportional to the strength of the effects; for events 1, 2, 3, 5 one circle = low effect < 25%; two circles = medium effect = between 25%–50%, three circles = high effect > 50%. Medium effect of CQ on proliferation means that actively proliferating cells are between 50%–75% with respect to untreated cells. For protein levels, due to the different effect of drugs in time-course, we have considered the mean of values obtained at 24 h and 48 h. For apoptosis (event 4), we used one black circle for <2 fold induction, and two black circles for > 2 fold induction. Moreover, parentheses [ ] indicate uncertain results due to high variability (as CQ effects on apoptosis and ALK expression).

By plotting together the effects produced by each drug treatment in terms of mRNA and protein levels, proliferation, and apoptosis, we observed that the efficacy of the three validated drugs was proportional to the level of PHOX2B mRNA reduced by treatments, and to the resulting protein amount (Figure 8A). Graphical representation of drug effects showed increasing effects in AA-CQ-MMF direction (Figure 8B), which can be expressed by linear functions (represented on the right of the diagram). Following the observations of the biological effects of the three drugs in terms of inhibition of proliferation and apoptosis induction we report here a grey-scale evaluation of the efficacy of AA, CQ, MMF, expressed as percentage of effect for each biological process compared to untreated cells, at least in one cell line (Figure 8C), where “NO” is for no effect, one circle is for effect less than 25%, two circles for effects between 25–50%, and three circles for marked effects (> 50%). Overall, this approach suggest us that the higher is the effect of drugs in decreasing *PHOX2B* mRNA amount, the stronger is their effect in downstream processes, in terms of protein amount, proliferation, apoptosis, and ALK expression.

## DISCUSSION

In this study, we report on two high throughput screenings (HTS) aimed at identifying drugs that are effective in counteracting neuroblastoma cell growth through the down-regulation of *PHOX2B* mRNA, whose excessive expression is likely to play a role in neuroblastoma pathogenesis [29], by targeting *PHOX2B* promoter and 3′ untranslated region. By applying several filters, we observed that the three drugs among the 6 selected compounds that resulted effective on endogenous mRNA are those acting on both the *PHOX2B* regulatory regions under analysis in HTS, showing their decrease in Luciferase reporter activity. Moreover, the present results, in agreement with those obtained from a previous study of ours [13], suggest that the threshold of Luciferase activity should be set below 50% normalized on untreated cells, as molecules displaying lesser effects have not been confirmed following the mRNA assay in both tests, likely due to the very high *PHOX2B* expression in neuroblastoma. Considering that mRNA levels are the consequence of the balance between transcriptional and post-transcriptional events, the remaining three drugs may act on other mechanisms that in our case could have counterbalanced the effects on *PHOX2B* promoter and 3′UTR, resulting in no change in the final mRNA levels. However, following treatments, a decrease in protein level was observed only for CQ and MMF, with different kinetics: in particular, while the effect of MMF on down-regulation of PHOX2B protein was detectable after 24 h and more markedly after 48 h treatment, CQ was able to down-regulate PHOX2B protein within 24 h, but after further 24 h the effect was no longer evident. This result could be ascribed to the function of CQ as inhibitor of autophagy, a cellular mechanism involved in elimination of mutant PHOX2B protein [30], whose inhibition by CQ could counterbalance its effect on *PHOX2B* mRNA levels. Moreover, as PHOX2B is overexpresseed in neuroblastoma, these results suggest that, to achieve down-regulation of the PHOX2B protein, it is necessary to select drugs that are very effective in reducing mRNA levels. We observed that vorinostat (SAHA), already selected from an epigenetic library in a previous smaller screening performed by us [13], and bortezomib, presently in phase I and II clinical trials for neuroblastoma, respectively ([31] http://clinicaltrial.gov), resulted to be very active in both HTS, thus confirming the reliability of our approach.

*PHOX2B* is down-regulated during the final steps of neuronal differentiation [9] and is involved in cell cycle exit [32]. Therefore, an alteration of *PHOX2B* levels could interfere with different stages of neuroblastoma development, starting from impaired differentiation of neuronal progenitors in the very early stages to their clonal expansion and tumor invasion. For this reason, we investigated whether *PHOX2B* down-regulation induced by the three selected drugs could decrease neuroblastoma cell growth by evaluating both apoptosis and cell proliferation. Apoptosis evaluation showed that only MMF was able to induce cell death, whereas AA did not show any effect and CQ variable effects were not statistically significant. However, both MMF and CQ resulted to be very efficient in decreasing neuroblastoma cell proliferation. These results are consistent with observations regarding mycophenolic acid, the active form of MMF and CQ, already reported to inhibit cell proliferation in gastric cancer cells [33] and in glioblastoma and lung tumor [34, 35], respectively.

Indeed, CQ is regarded as a novel anti-tumor agent, acting on apoptosis or cell cycle arrest, depending on the cellular models [25]. Very recently, CQ has been proposed as adjuvant in chemotherapy for neuroblastoma as its pro-autophagic role seemed to enhance the efficacy of the ALK inhibitor entrectinib [24]. Though it has been mainly used so far for its effect in regulating the autophagy process, our HTS results confirm CQ as a specific anti- neuroblastoma agent acting through *PHOX2B* down-regulation, and therefore indicate CQ as an effective key molecule in neuroblastoma treatment. Finally, the effect of vorinostat and bortezomib, both in clinical trials for neuroblastoma [31], proved to be enhanced by CQ in a model of colon cancer [36] and in lymphoma cells [37].

MMF has been reported to decrease cell proliferation not only in tumor, as in human conjunctival goblet cells (CGCs) [38]. In neuroblastoma, MMF has been observed to induce differentiation, apoptosis, and cell cycle arrest through a p53-mediated pathway [26, 38]. Taken together, our data on *PHOX2B* gene expression and neuroblastoma cell proliferation and apoptosis showed that MMF and CQ are the most effective drugs among those selected through HTS in counteracting neuroblastoma. *In vivo* and *in vitro* studies on PHOX2B role inneuroblastoma yield different results.

The knockdown of PHOX2B in human neuroblastoma micrometastatic cells proved to increase with tumorigenic and metastatic potential [13]. However, down-regulation of mature neuronal genes, including *PHOX2B*, arrested the differentiation of malignant human neuroblastoma SH-SY5Y cells and enhanced their sensitivity to anticancer drugs [39]. These differences could have several explanation, for instance the modulation of *PHOX2B* gene has been shown to led to distinct consequences depending on the embryonic stage of neural development, as in the case of the effect of the expression of PHOX2BΔ8 mutation on the correct development of locus coeruleus [40]. Moreover, PHOX2B gene dosage is important for the correct development of the sympathetic neuronal system, as shown in a zebrafish model of neuroblastoma where both *phox2b* knockout and *phox2b* neuroblastoma associated mutation led to a block of differentiation [41]. Therefore, *PHOX2B* gene down-regulation in the early phase of tumorigenesis may affect tumor differentiation and growth, without affecting the late metastatic cancer stage.

We could postulate that both too much low and too much high expression are damaging and that CQ and MMF may be effective in rendering neuroblastoma cells more sensitive to pro-apoptotic signals. Overall, the results of this study suggest that MMF and CQ could be considered for neuroblastoma therapy, as they displayed specific effects on neuroblastoma cell viability mediated by PHOX2B gene expression down-regulation. Results reported here fall within the second phase of an ideal drug discovery process, consisting in the “hit” screening. At this stage, MMF and CQ have not been validated for neuroblastoma therapy yet, however our work provides experimental data recommending further processes of MMF and CQ validation. In particular, due to the biological heterogeneity of neuroblastoma in CQ response, demonstrated by results on apoptosis in the two cell lines used, further validations in animal models should be performed to definitely confirm a role for this drug also in neuroblastoma.

## MATERIALS AND METHODS

### Production of the “pGL4.17PHOX2B-promoter” stable cell line

IMR32 cells were transfected with 3 μg pGL4.17-PHOX2B promoter plasmid, in which the *luciferase* gene was under the control of 1 Kb region upstream the *PHOX2B* coding region (150 bp 5′UTR + 850 bp promoter), and was grown under G418 selection, as previously described [14].

### Production of the “pmiRGlo-PHOX2B-3′UTR” and “pmiRGlo(empty)” stable cell lines

IMR32 cells were plated in 10 mm dishes at 60% confluence and transfected with 3 μg of the pmiRGlo-3′UTR *PHOX2B* vector, containing the 3′UTR full length and generated as previously described [16] or, alternatively, with the pmiRGlo(empty) vector, lacking the *PHOX2B* 3′UTR sequence. After 24 h, the transfected cells were grown in RPMI medium added with 450 μg/ml G418. The pmiRGlo vector backbone contains the coding regions of both the firefly *luciferase* gene (whose expression depends on the upstream PGK promoter, a downstream polyA signal and the presence in between of the *PHOX2B* 3′UTR region) and the renilla *luciferase* gene fused to the neomycin gene (whose expression depends on the SV40 promoter and a downstream polyA signal). Therefore, both firefly and renilla Luciferase activities (Dual Luciferase reporter assay system, Promega; TD 20/20 luminometer) were evaluated and only clones positive for both values were considered suitable for further experiments.

### Preparation and use of the FDA library

The Screen-Well^®^ FDA approved drug library (version 1.5, Enzo Life Sciences, BML-2842-0100) contains 640 compounds with known and well-characterized bioactivity, safety, and bioavailability. It is a collection of molecules suitable for drug repurposing aims, therefore drugs were carefully selected to maximize chemical and pharmacological diversity. Molecules are provided in 8 multiwell plates at 10 mM concentration in DMSO. In each plate, the four wells of the first and last columns were filled with the following samples: only medium (no cells), DMSO, trichostatin A (TSA) (positive control) and triacetyl-resveratrol (negative control) [14]. Serial dilutions of each plate, starting from the 10mM stock supplied, were produced in order to have a final working dose of 5 μM in 0.5% DMSO.

### High throughput screening (HTS) of drugs

To perform the automated drug screening, 30–40,000 cells were grown in white multiwell plates, added with drugs, and analyzed 24 h later. As the screening of the *PHOX2B* 3′UTR was performed in this study for the first time, no positive or negative control was known. Therefore, while the *PHOX2B* promoter screening (promoter HTS) was conducted in duplicate and in the presence of positive/negative controls, to obtain the most reliable screening even in the absence of controls, the 3′UTR screening (3UTR HTS) was carried out in triplicate. Both the luminescence produced by the firefly *luciferase* gene and the fluorescence produced by protease-mediated activity on the glycylphenylalanyl-aminofluorocoumarin (GF-AFC) substrate in living cells were measured by using a two-step assay which yielded the double information in a single well (ONE-Glo^™^ + Tox Luciferase Reporter and Cell Viability Assay, Promega). Either parameters were measured by an automated microplate reader (GloMax^®^ Promega) following an already set up procedure [14]. To avoid misleading results that might be ascribed to the effect of drugs on the regulatory regions upstream or downstream the renilla gene, in the 3UTR HTS, renilla firefly activity was not measured.

### Quality evaluation of HTS

A total of 16 plates were used to conduct the “promoter HTS”. Signals were corrected for background as determined in wells with the medium only. Z′-factors were calculated for each plate in PHOX2B *promoter* HTS, using the formula $Z^{\prime }=1-\frac{3\sigma_{c+}+3\sigma_{c-}}{\left|\mu_{c+}-\mu_{c-}\right|}$, [42] where σ_c+_ and σ_c-_ are the standard deviation values of positive and negative samples and μ represents their average value. For the PHOX2B *promoter* screening Z’ factor was 0.8011(± 0.1226), a value corresponding to an excellent assay. A total of 32 plates were used in the 3′UTR HTS, as it was performed in triplicate. In this case, we calculated the Z-factor, that evaluates the quality of samples compounds tested by comparing the mean of all compounds analyzed with the mean value of non treated cells $Z=1-\frac{3\sigma_{S}+3\sigma_{c-}}{\left|\mu_{S}-\mu_{c-}\right|}$, where (σ) is the standard deviation and *(μ* ) is the mean signals for negative (c-) and sample wells *(s). Z-factor*, as a value of the HTS quality, with a had an average score of 0.49 (± 0.108) that indicates a good assay.

### Preparation of drug for validation assays

Lovastatin (Enzo Life Sciences), Tranylcypromine (Enzo Life Sciences), Chloroquine Phosphate (CQ, Enzo Life Sciences), Acetylsalicylic Acid (AA, Sigma-Aldrich), Sulindac (Enzo Life Sciences), Mycophenolate Mofetil (MMF, Enzo Life Sciences), and Amifostine (Enzo Life Sciences) were prepared for validation as a stock solution in DMSO or H_2_O and dilutions were produced to obtain working concentrations in cell culture medium.

### Gene expression analysis

To evaluate the effect of drug treatments on *PHOX2B* and *ALK* expression, 2 × 10^5^ IMR-32 cells were plated in multiwell plates (6-wells) 24 h prior to treatment. At the end of treatment, the total cellular RNA was extracted and purified with RNeasy Plus mini kit (Qiagen) and the RNA samples thus obtained were quantified by NanoDrop (Thermo Scientific, Rockford, USA). cDNA was synthesized from 1 μg of total RNA by using the iScript Reverse Transcription Supermix for RT-qPCR, (Biorad), according to the manufacturers’ protocol. Analysis and quantification of *PHOX2B* and ALK mRNA was performed by a two-step RT-qPCR (iQ5 BioRad) using TaqMan Assay probes (PHOX2B: ID Hs00243679_m1, ALK: ID Hs00608289, Life Technologies). Reactions were carried out in 20 μl total volume with 10 μl of 2x TaqMan IQ SuperMix (BioRad) and 1 μl of 20x specific assays; the thermocycler protocol was as follows: initial denaturation at 95°C for 2 min, 40 cycles at 95°C for 15 sec and 60°C for 30 sec. Three independent biological replicates were performed for each treatment to minimize manual variability. In each experiment, *b2-microglobulin*(b2m, ID Hs99999907_m1; Life Technologies) and *G3PDH* (ID Hs99999905_m1; Life Technologies), whose expression among several tested housekeeping genes resulted not to be affected by drug treatments, were used as reference genes for data normalization. Data were processed with the BioRad IQ5 software, using the ddCT method.

### Western blot assay

3 × 10^5^ IMR-32 cells were grown in a 6-multiwell plate and, 24 h and 48 h after treatments, they were washed with PBS 1×, centrifuged, and treated with RIPA buffer (Tris–HCl 50 mM pH 7.5, NaCl 150 mM, Triton-X 1%, SDS-20 0.1%, Na deoxycholate 1%, Protease Inhibitor mix 1×). Total cell lysates were quantified (Quick Start Bredford 1X Dye Reagent, BioRad) and equal amounts were electrophoresed on 10% SDS-PAGE and transferred onto a polyvinylidene difluoride membrane (Millipore). Chicken PHOX2B antibody [43], goat anti-chicken HRP antibody (Santa Cruz Biotechnology), mouse anti- β- tubulin antibody (Sigma-Aldrich), goat anti mouse HRP antibody (Dako) were used. Signals were detected using the ECL advance chemiluminescence reagent (Amersham). Images were acquired by UVITEC Alliance Mini HD9 Touch (Eppendorf) and quantification of the detected protein levels was performed by UVITEC NineAlliance 1D software.

### Morphological evaluation of nuclei

150.000 IMR32 cells were plated in amniodishes (Euroclone) and added with drugs for 48 hours followed by nuclear DAPI staining. Apoptosis was investigated by fluorescence microscopy in terms of nuclear shape, such as fragmentation into many small, intensely fluorescent homogeneous bodies. The percentage of aberrant nuclei was obtained by counting all acquired nuclei and by calculating the ratio of disrupted to total nuclei for each condition.

### Proliferation and apoptosis evaluation

Human neuroblastoma IMR32 and HTLA-230 cancer cells were plated in 12 well plates (Falcon, Becton Dickinson, Le Pont de Claix, France) (1,000,000 cells/well) in DMEM media (Euroclone, Milan, Italy) supplemented with 10% Fetal Bovine Serum (FBS) and 1% L-glutamine and P/S. After 24 hours, cells were treated with Chloroquine (CQ, 400 nm), Mycophenolate mofetil (MMF, 5 μM), and Acetilsalicylic acid (AA, 5 μM). Forty-eight hours later, cells were harvested and labeled with CarboxyfluoresceinSuccinimidyl ester (CFSE) (Invitrogen, Milano, Italy) or AnnexinV/Propidium Iodide (eBioscience, Milan, Italy) to assess proliferation and apoptosis, respectively. Then, cells were acquired with the Gallios cytometer (Beckman Coulter, Milan, Italy)

### Analysis of caspases 3/7 activity

The activity of caspases was measured by a Luciferase reporter-based approach (Caspase-Glo^®^ 3/7 Assay (Promega). In detail, 25,000 cells were seeded in a white multiwell plate and, after 24 hours, 100 μl culture medium containing either drug or solvent was added. Forty-eight hours after treatment, an equal volume of Caspase-Glo^®^ Reagent was added to each well, including empty wells containing only medium, in order to assess the specificity of the assay. After 2 hours incubation, the luminescence produced by the luminogenic caspase-3/7 substrate was measured using the GloMax^®^ 96 Microplate Luminometer (Promega). For each sample, the experiment was performed in triplicate and repeated three times.

### Statistical analysis

Student's *t* test and ANOVA test were performed for treated and untreated samples. Moreover, the R^2^, a measure of the strength of linear regression for each variable, was calculated.

## SUPPLEMENTARY FIGURES



## Abbreviations

PHOX2B: Paired-like homeobox 2b
ALK: Anaplastic Leukemia Kinase
AA: acetylsalicylic acid
CQ: chloroquine phosphate
MMF: mycophenolate motefil
3′UTR: 3′ untranslated region
FDA: Food and Drug Administration
HTS: high throughput screening
CFSE: Carboxyfluorescein Diacetate Succinimidyl Ester
MFI: mean fluorescence intensity
PI: propidium iodide
RLU: Relative Light Unit
TUB: tubulin
