# Supplementary material for: Targeting of PHOX2B expression allows the identification of drugs effective in counteracting neuroblastoma cell growth
Source: Oncotarget. 2017 Aug 4;8(42):72133–46. doi: 10.18632/oncotarget.19922 (PMC5641117; doi:10.18632/oncotarget.19922)
Supplement: Supplementary file 1 [file oncotarget-08-72133-s001.pdf]

Targeting of *PHOX2B* expression allows the identification of drugs effective in counteracting neuroblastoma cell growth

SUPPLEMENTARY MATERIALS

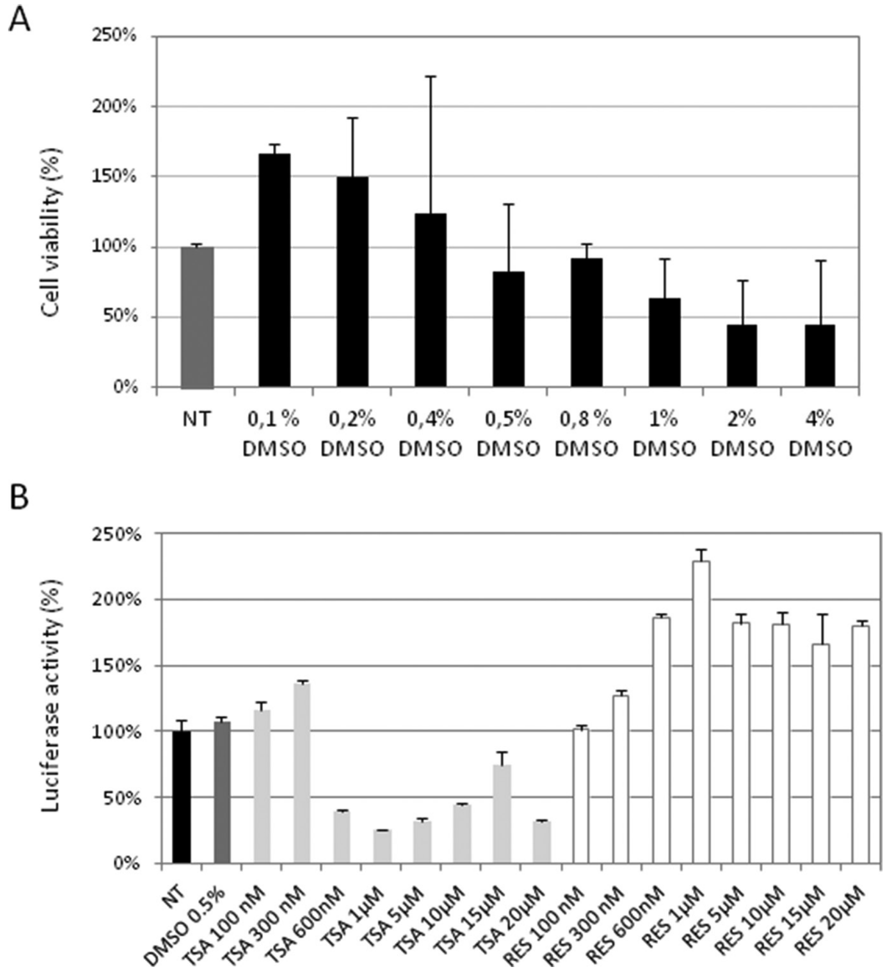

Supplementary Figure 1: Set up of HTS experimental conditions. (**A**) Cell viability has been evaluated in terms of AFC fluorescence in the IMR32 "*PHOX2B* promoter" clone after addition of increasing doses of DMSO for 24 hours. (**B**) Luciferase activity driven by the *PHOX2B* promoter has been measured in a dose-response curve following treatments for 24 hours with progressively increasing doses of the positive control drug trichostatin A (TSA) and the negative control drug tryacilresveratrol (RES).

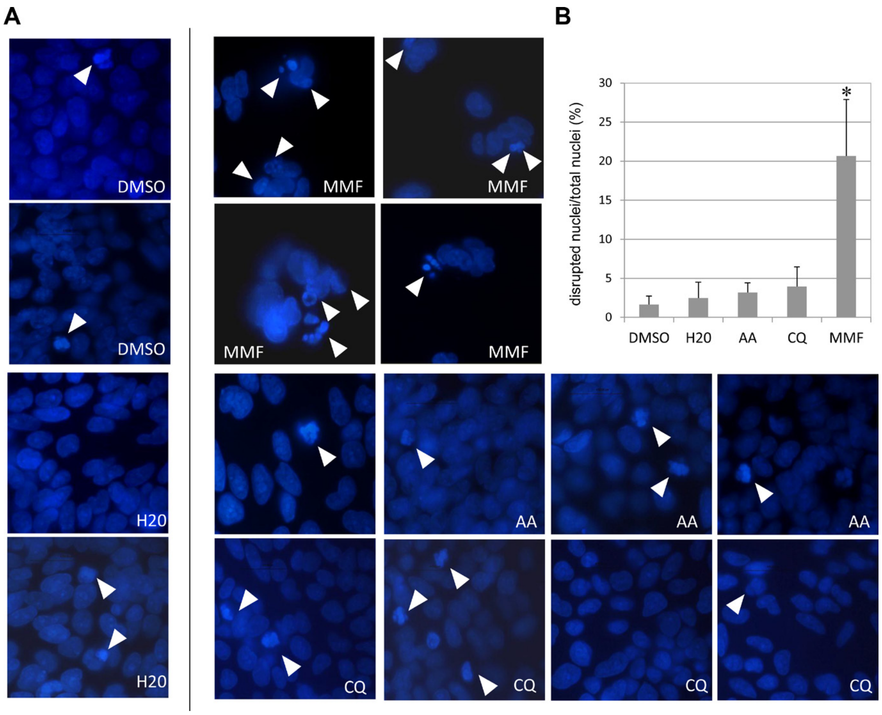

Supplementary Figure 2: Analysis of nuclear morphology after drugs treatments. (**A**) Fluorescence microscope analysis of nuclear morphology after 48 treatments with MMF, CQ, and AA and their diluents H_2O and DMSO. Arrows indicate disrupted nuclei or nuclei with aberrant borders or vacuoles, suggestive of apoptosis. In particular, in MMF treated samples, very few adherent cells can be detected after 48 h treatment. (**B**) The percentage of apoptotic nuclei out of total nuclei was reported in the bar diagram; statistical significance is indicated by * ($N = 3$; Student *t* test, $p = 0.018$).

IMR32

early / late

AA — early

source	df	SS	MS	F	P-value
treatments	1	0.167	0.167	0.5000	0.8381
error	4	1.333	0.333		
total	5	1.500			

AA — late

source	df	SS	MS	F	P-value
treatments	1	0.000	0.000	0.0000	1.0000
error	4	1.333	0.333		
total	5	1.333			

CQ — early

source	df	SS	MS	F	P-value
treatments	1	13.500	13.500	4.0500	0.3503
error	4	13.333	3.333		
total	5	26.833			

CQ — late

source	df	SS	MS	F	P-value
treatments	1	20.167	20.167	7.1176	0.2158
error	4	11.333	2.833		
total	5	31.500			

MMF — early

source	df	SS	MS	F	P-value
treatments	1	0.000	0.000	0.0000	1.0000
error	4	1.333	0.333		
total	5	1.333			

MMF — late

source	df	SS	MS	F	P-value
treatments	1	24.000	24.000	72.0000	0.0121
error	4	1.333	0.333		
total	5	25.333			

HTLA-230

early / late

AA — early

source	df	SS	MS	F	P-value
treatments	1	2.667	2.667	1.1429	0.6859
error	4	9.333	2.333		
total	5	12.000			

AA — late

source	df	SS	MS	F	P-value
treatments	1	0.533	0.533	2.4000	0.5099
error	3	0.667	0.222		
total	4	1.200			

CQ — early

source	df	SS	MS	F	P-value
treatments	1	8.167	8.167	3.0625	0.4262
error	4	10.667	2.667		
total	5	18.833			

CQ — late

source	df	SS	MS	F	P-value
treatments	1	0.133	0.133	0.6000	0.8162
error	3	0.667	0.222		
total	4	0.800			

MMF — early

source	df	SS	MS	F	P-value
treatments	1	4.167	4.167	1.9231	0.5550
error	4	8.667	2.167		
total	5	12.833			

MMF — late

source	df	SS	MS	F	P-value
treatments	1	0.133	0.133	0.6000	0.8162
error	3	0.667	0.222		
total	4	0.800			

Supplementary Figure 3: Statistical analysis of apoptosis in IMR32 and HTLA-230 treated with CQ and MMF. One-way analysis of variance (ANOVA) table including sums of squares (SS), degrees of freedom (df), mean squares (MS), and F and p-values, given the mean, standard deviation, and number of subjects in each group. Results obtained for early and late apoptosis in IMR32 and HTLA-230 following the three treatments are presented in the upper and lower part of the figure, respectively. The statistical significance in indicated by a red box.

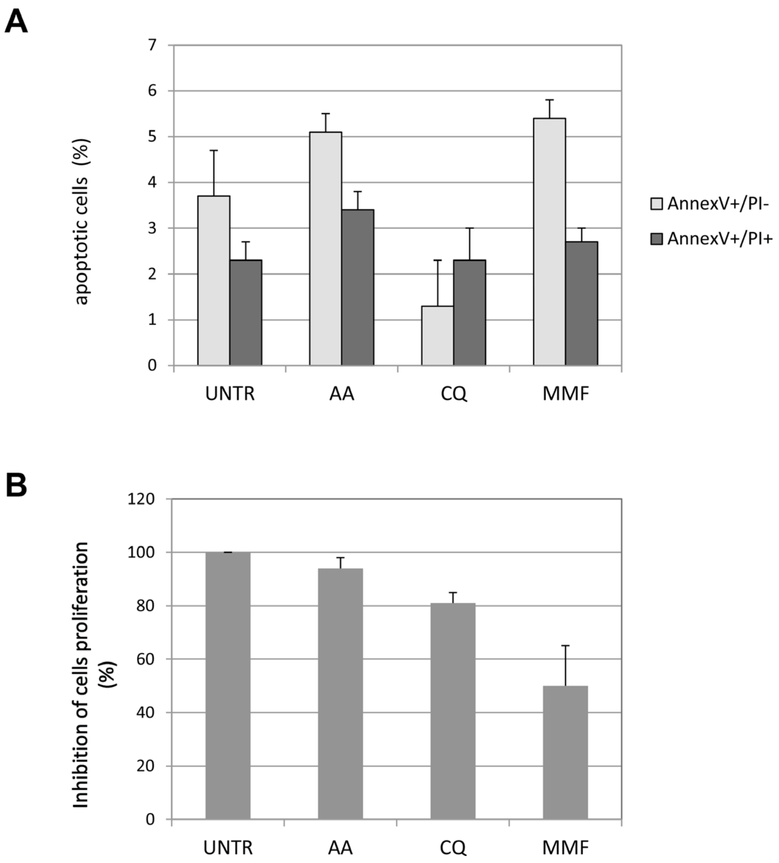

Supplementary Figure 4: Evaluation of effects of drugs on apoptosis and cell proliferation in HTLA-230 cells. (**A**) Staining of HTLA-230 cells by flow cytometry with Annexin V/PI to evaluate apoptosis following 48 h drugs treatments. the early and late apoptotic cells, expressed as percentage out of the total number of cells. (**B**) Cell proliferation rate after drugs addition for 48 h is shown as percentage of proliferation calculated as 100-percentage of inhibition (obtained as MFI of CFSE), based on observation that an increase in MFI indicates inhibition of cell proliferation, as dividing cells contain half fluorescent dye. Values are the mean of three independent samples ± SD; asterisks indicate significant differences compared to untreated cells.
